# Does the Australian influenza season predict the Canadian influenza season? A qualitative comparison of seasons, 2014–2020

**DOI:** 10.14745/ccdr.v49i1112a05

**Published:** 2023-11-01

**Authors:** Deborah Chan, Liza Lee, Christina Bancej

**Affiliations:** 1 Centre for Immunization and Respiratory Infectious Diseases, Public Health Agency of Canada, Ottawa, ON

**Keywords:** influenza, surveillance, seasonality, epidemiology, vaccine

## Abstract

A commonly held belief by the Canadian media and public is that the Australian influenza season is a fairly reliable indicator of what the Canadian influenza season that follows might be like. However, this claim is not well substantiated with epidemiological evidence. Therefore, the objective of this work was to qualitatively compare the timing of the onset, peak, and intensity of influenza activity, the dominant circulating influenza strains, and the seasonal vaccine and vaccination policies from 2014 to 2020 between Canada and Australia, using a combination of FluNet data and influenza surveillance reports and publications. Across the epidemiological indicators considered, the epidemics between Canada and Australia often differ. While vaccination policies and coverage are similar between the two countries, vaccine composition and vaccine effectiveness estimates also differ. Ultimately, there are many differences and confounding variables between the Australian and Canadian influenza seasons across numerous indicators that preclude the use of the Australian influenza season as the sole predictor of the Canadian influenza season. However, the availability of global surveillance data and robust national and sub-national surveillance data can provide lead time and inform within-season resource and capacity planning, as well as mitigation measures, for seasonal influenza epidemics.

## Introduction

Seasonal influenza primarily circulates in the winter months. In Australia, the influenza season typically occurs during the months of May to October, while in Canada, the influenza season typically occurs during the months of October to May. A commonly held belief by the Canadian media and public is that the Australian influenza season is a fairly reliable indicator of what the Canadian influenza season that follows might be like ((1–4)) The origin of this belief is unknown, but likely became widespread after the severe influenza seasons in both the Southern and Northern Hemispheres in 2017 ((2)).

As of December 2021, only one empirical study has been published on whether Australian influenza data can predict influenza activity in the Northern Hemisphere (the United States, United Kingdom, and China). Zhang *et al.* applied a multivariate seasonal autoregression integrated moving average model and found that using World Health Organization (WHO) FluNet surveillance data from 2010 to 2018 for the Southern Hemisphere, in combination with local data from internet queries, nominally improved prediction of the influenza positive incidence in these three Northern Hemisphere countries ((5)). Beyond this, the claim that the Australian influenza season can be used to predict the Canadian influenza season is not well investigated nor substantiated by epidemiological evidence.

The objective of this commentary is to compare the timing of the onset, peak, and intensity of influenza activity, the dominant circulating influenza strains, and the seasonal vaccine and vaccination policies from 2014 to 2020 between Canada and Australia to determine whether there is sufficient evidence to support whether the seasonal influenza epidemic in Australia can be used as a predictor of the Canadian influenza season.

## Scope and methods

Data from seven consecutive seasons (Northern Hemisphere seasons 2014–2015 to 2020–2021 and corresponding Southern Hemisphere seasons 2014 to 2020) were used for qualitative comparison. The WHO FluNet data were used to determine the dominant circulating subtype and to calculate and generate the influenza A and B percent positivity epidemiological curves for Australia and Canada from January 2014 to August 2021 ((6)). To enable the comparison of seasons, start and end, epidemiological week 35 of a Canadian season was aligned to week 1 of an Australian season and periods. Australia does not set a threshold to call the start and end to their seasonal epidemic; therefore, to enable a direct comparison, Canada’s threshold of two consecutive weeks of ≥5% influenza test positivity was used to define a seasonal influenza epidemic (7). Epidemiological curves were compared and analyzed. All analyses were done in R software ((8)) and figures were produced in Excel.

Hospitalizations, while an important surveillance indicator for severity, were excluded from this comparison, as hospitalization data between the two countries were not comparable. Data used for the comparison of epidemiological trends and vaccination recommendations were limited to official surveillance reports and immunization handbooks and statements published by the Government of Canada and the Australian Government. Information on seasonal influenza vaccine composition was obtained from the meeting reports published by the WHO. Vaccine effectiveness (VE) results were obtained from published journal articles that were collected, collated and saved as part of active surveillance of global VE results by Canada’s national influenza surveillance program (FluWatch).

## Key findings

### Virologic

Influenza A was the dominant circulating virus type in both Canada and Australia across seasons, with the exception of the 2015 season in Australia, where influenza A and B circulated in similar proportions (**Table 1**). Over the seven seasons compared, in only three did the dominant Australian influenza A subtype correspond to the following season’s dominant Canadian influenza A subtype (2016/2016–2017 [A(H3N2)], 2017/2017–2018 [A(H3N2)] and 2018/2018–2019 [A(H1N1)] seasons). While strain information on the influenza A subtypes in circulation were unavailable in the Australian surveillance reports, dominant influenza A subtype in circulation in Australia during the three seasons were determined to be well matched, reasonably well matched, or antigenically similar to the vaccine components, respectively (9–11). This suggests that the dominant circulating strains of influenza A subtypes were similar to those in Canada during these three seasons (12–14).

**Table 1:** Number and proportion of Influenza detections by type, Australia and Canada, 2014–2020^a^

Season	Country	Total influenza detections	Influenza A		Influenza B		Among subtyped influenza A detections			
							Influenza H1N1		Influenza H3N2	
			n	%	n	%	n	%	n	%
2014/2014–2015	Australia	3,473	**3,011**	**86.7**	462	13.3	**1,701**	**60.2**	1,124	39.8
	Canada	45,048	**36,428**	**80.9**	8,620	19.1	104	0.8	**13,168**	**99.2**
2015/2015–2016	Australia	3,625	1,825	50.3	1,800	49.7	244	13.7	**1,533**	**86.3**
	Canada	39,449	**28,495**	**72.2**	10,954	27.8	**11,168**	**90.5**	1,172	9.5
2016/2016–2017	Australia	6,705	**5,566**	**83.0**	1,139	17.0	588	16.9	**2,893**	**83.1**
	Canada	39,512	**35,001**	**88.6**	4,511	11.4	176	1.0	**17,524**	**99.0**
2017/2017–2018	Australia	10,509	**7,684**	**73.1**	2,825	26.9	507	18.4	**2,248**	**81.6**
	Canada	64,250	**36,039**	**56.1**	28,211	43.9	1,274	10.3	**11,074**	**89.7**
2018/2018–2019	Australia	4,264	**3,869**	**90.7**	395	9.3	**2,058**	**74.8**	695	25.2
	Canada	47,763	**45,240**	**94.7**	2,523	5.3	**10,981**	**67.9**	5,196	32.1
2019/2019–2020	Australia	14,002	**12,035**	**86.0**	1,967	14.0	674	12.8	**4,586**	**87.2**
	Canada	53,789	**30,986**	**57.6**	22,803	42.4	**4,956**	**69.1**	2,215	30.9
2020/2020–2021	Australia	949	**876**	**92.3**	73	7.7	**267**	**80.7**	64	19.3
	Canada	72	**49**	**68.1**	23	31.9	5	38.5	**8**	**61.5**

^a^ Dominant circulating type and influenza A subtype by country and season are indicated in bold

**Figure 1:**
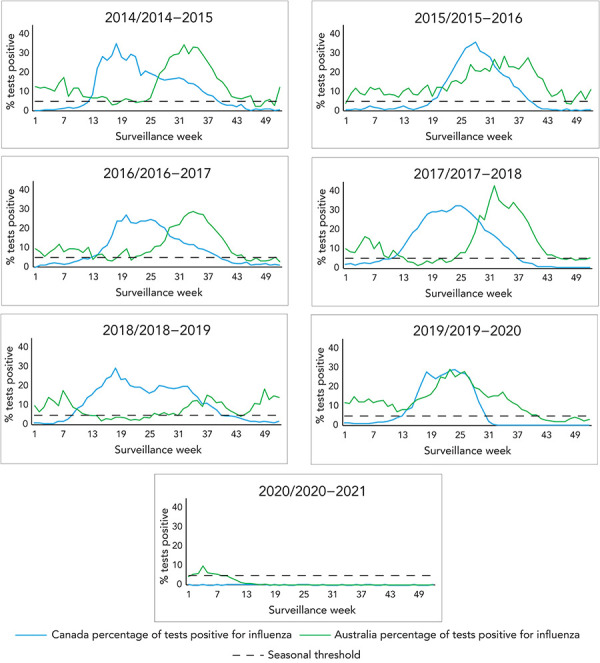
Historical comparison of influenza percent positivity in Canada and Australia, by surveillance week and season

During this period, both Canada and Australia had seasons with influenza B circulation, but the seasons with higher influenza B incidence had no correspondence (2015 in Australia vs. 2017–2018 and 2019–2020 in Canada). Across most seasons, Canada had a large wave of influenza A followed by a smaller wave of influenza B, except in seasons 2017–2018 and 2019–2020, where influenza B co-circulated with influenza A (**Figure 1**). In Australia, influenza A and B generally co-circulated in all seasons, with influenza B circulating at lower levels. Due to the coronavirus disease 2019 (COVID-19) pandemic and public health response measures, both Australia and Canada had minimal circulating influenza in 2020–2021.

### Influenza activity

**Figure 2:**
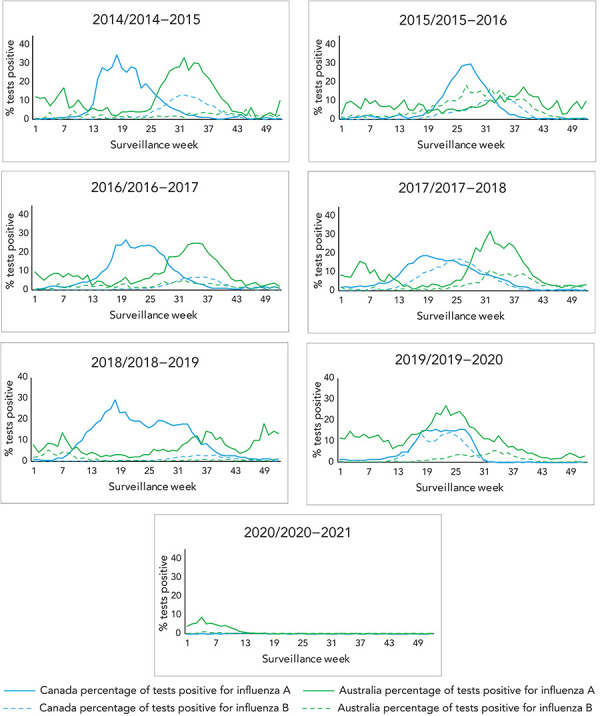
Historical comparison of influenza A and B percent positivity in Canada and Australia by surveillance week and season

Australia and Canada also show different seasonal dynamics and differ from one season to another (**Figure 2**). Using Canada’s thresholds for seasonal influenza epidemics (at least two consecutive weeks where ≥5% of tests are positive for influenza) as a marker for epidemic activity, Australia appears to experience a short and less intense epidemic period of influenza activity in most seasons before experiencing the main, larger epidemic, while Canada usually experiences one continuous period of epidemic activity. Excluding the Canadian 2019–2020 and 2020–2021 seasons and the Australian 2020 season due to the COVID-19 pandemic, the average epidemic length in Canada was 27 weeks (range: 22–31 weeks) and 31 weeks in Australia (range: 23–45 weeks). Excluding the seasons affected by the COVID-19 pandemic, Australia sees an average of 40 weeks where at least 5% positivity was reported, compared to Canada’s average of 27 weeks.

Epidemic activity rises more quickly in Canada (with seasons peaking on average during? 10.4 weeks in Canada [range: 8–15 weeks] vs. 17 weeks for Australia [range: 9–33 weeks]) from the time where 5% positivity is reached in the main epidemic curve. The intensity, as indicated by the magnitude of the peak, differed between Canada and Australia for most seasons. There was only one season (2016/2016–17), where the peak percent positivity in Canada and Australia was within 5%. There was no discernable peak pattern, as peak percent positivity ranged from 15.1%–42.9% in Australia and 27.1%–36.0% in Canada.

### Vaccine policy and coverage

Vaccine policies are relatively similar between Australia and Canada. The Australian Immunisation Handbook and the Canadian Immunization Guide both outline similar groups recommended for seasonal influenza vaccination. For both countries, all individuals aged ≥6 months should be offered the seasonal influenza vaccine with a focus on groups that include individuals at high risk of influenza-related complications or hospitalization, individuals capable of transmitting influenza to those at high risk, individuals who provide essential community services and commercial poultry (both Canada and Australia), and swine workers (Australia only) during an outbreak of avian or swine influenza ((15,16)).

In the 2020/2020–2021 season, vaccine coverage in both countries was also relatively similar. Vaccine coverage was highest among individuals aged 65+ (62% in Australia and 70% in Canada) (17,18). Adults also had similar coverage in both countries (in Australia 23% and 35% of individuals aged 15 to ≤49 years and 50 to ≤64 years respectively were vaccinated and in Canada, 29% in individuals aged 18–64 years in Canada) (17,18). Vaccination coverage is relatively stable year to year in both countries.

### Influenza vaccine composition and vaccine effectiveness

Vaccine strain recommendations were identical between Australia and Canada from 2014 to 2017, with both countries providing both trivalent and quadrivalent vaccines. The recommended B strains differed in 2018, 2019 and 2020 and A strains differed in 2019 and 2020 (19).

Vaccine effectiveness estimates generated using similar test negative case control designs for comparable seasons and intervals are summarized in **Table 2**. For three out of the four seasons where the vaccines were identical, Australia’s VE estimate was higher than that of Canada’s (with the exception of the 2016–2017 season); however, the confidence intervals overlapped in all but the 2014–2015 season, where the VE in Canada was 9% vs. 44% in Australia).

**Table 2:** Summary of published vaccine effectiveness estimates (interim or final) against medically attended influenza, Australia and Canada, seasons 2014 to 2020

Season (references)	Australia VE estimate (95% CI)	Canada VE estimate (95% CI)	Notes on VE estimate^a^
2014/2014–2015 ((20,21)	44% (31–55)	9% (−14 –57)	VE against medically attended influenza (all types)
2015/2015–2016 ((22,23))	54% (42–63)	46% (32–57)	VE against medically attended influenza (all types)
2016/2016–2017 ((24,25))	40% (18–56)	44% (30–55)	VE against medically attended influenza (all types)
2017/2017–2018 ((26,27))	55% (17–46)	42% (25–55)	Interim VE against medically attended influenza (all types)
2018/2018–2019^b^ (28,29)	68% (47–67)	68% (55–77)	Interim VE against medically attended influenza (all types)
2019/2019–2020^c^ (30,31)	A(H1N1): 62% (39–78) A(H3N2): 37% (24–49) B: 63% (45–74)	A(H1N1): 44% (26–58) A(H3N2): 62% (37–77) B: 69% (57–77)	Interim VE against medically attended influenza (by type/subtype)
2020/2020–2021^d^	N/A	N/A	N/A

Abbreviations: CI, confidence interval; N/A, not applicable; VE, vaccine effectiveness

^a^ The most up-to-date comparable estimates available were used: If only interim estimates were available for one country, the interim estimates for both countries were used for the comparison

^b^ The 2018 Southern Hemisphere and 2018–2019 Northern Hemisphere vaccine had a different influenza B Victoria component

^c^ The 2019 Southern Hemisphere and 2019–2020 Northern Hemisphere vaccine had different influenza A(H1N1) and A(H3N2) components

^d^ The 2020 Southern Hemisphere and the 2020–2021 Northern Hemisphere vaccine had different influenza A(H1N1) and A(H3N2) components

## Discussion

Australia and Canada have different seasonal dynamics and overall activity differs from one season to another. The Canadian influenza season appears to be more concentrated with activity peaking more quickly than that of the Australian influenza season. The dominant circulating type and subtype can have an effect on the burden and severity of a season. The dominant circulating type and subtype, length, intensity and activity of an influenza season are core surveillance indicators in Canada. Our comparison showed that these indicators are often different between the countries from season to season.

Vaccine policy and coverage are similar between the countries and among the seasons, with comparable vaccine components. No distinct VE estimate trends were found between the two countries. More recently, the composition of the Northern and Southern Hemispheres seasonal influenza vaccine began to differ, which limits the comparability and usefulness of the Australian VE estimates as a predictor of Canadian VE estimates. Differences and similarities in vaccine composition, policy and VE are other limitations that must be considered when comparing the influenza activity of the two countries and using one as a predictor of activity for the other.

In addition to the differences in seasonal activity, climatic and demographic factors are well-established factors that influence influenza disease dynamics ((32,33)). Both similarities and differences exist between Canada and Australia in climate and population. The climate of the two countries is different, with sub-zero degrees Celsius winter temperatures in Canada and above zero degrees Celsius in Australia. The population distribution, however, in 2020 by age and sex are similar between the two countries ((34,35)).

Confounding issues in the side-by-side analysis of standard surveillance indicators is a major limitation of this analysis. For example, laboratory-confirmed influenza is a nationally notifiable disease in both Australia and Canada; however, there can be differences in the populations being tested and testing practices between the countries. This is evidenced by the differences in the number of influenza detections reported between Canada and Australia. In some seasons, Canada has greater than 10 times the influenza detections; however, it is unknown whether this is due to differences in testing and reporting practices or actual differences in the number of detections (illness). Canada leans towards testing more severe disease in patients; however, Australia’s testing strategy may differ from Canada. The metadata to assess data comparability and potential threats to validity are often unavailable in routine surveillance reports or from the underlying surveillance systems.

Influenza activity is notoriously hard to predict. The attraction of using the seasonal influenza experience that occurred just months before in one country to predict the activity of another country is understandable from a planning perspective. The Australian influenza surveillance reports are available online and they have robust surveillance indicators; however, there are many important considerations outlined in this article that should be taken into account when interpreting the data and applying it to Canada.

## Conclusion

This comparison is the first season-by-season comparison of Canadian and Australian influenza data to our knowledge, and it brings to light the challenges and limitations with using Australia’s data to predict Canada’s influenza season. Based on this comparison, the use of key indicators from the Australian season to predict trajectory, intensity or duration characteristics of the Canadian influenza season is unsupported by evidence. While we are not discounting the use of Australian influenza surveillance data, the data should be treated the same way as surveillance data obtained from any other country and used together as global intelligence to inform influenza trends and activity that could occur in Canada. Timely and robust national and sub-national surveillance data is a great asset in aiding the development of within-season predictions that can provide lead time and inform within-season resource and capacity planning, as well as mitigation measures ((36)).
